# Temporal Change in Alert Override Rate with a Minimally Interruptive Clinical Decision Support on a Next-Generation Electronic Medical Record

**DOI:** 10.3390/medicina56120662

**Published:** 2020-11-30

**Authors:** Won Chul Cha, Weon Jung, Jaeyong Yu, Junsang Yoo, Jinwook Choi

**Affiliations:** 1 Department of Digital Health, SAIHST, Sungkyunkwan University, Seoul 06355, Korea; wc.cha@samsung.com (W.C.C.); angela.weon@gmail.com (W.J.); icalust@naver.com (J.Y.); 2 Department of Biomedical Engineering, Seoul National University College of Medicine, Seoul 03080, Korea; 3 Department of Emergency Medicine, Samsung Medical Center, Seoul 06355, Korea; 4 Department of Nursing, Sahmyook University, School of Nursing, Institution of Healthcare Resource, Seoul 01795, Korea; junnsang@gmail.com

**Keywords:** decision support systems, clinical, electronic health records, medical order entry systems, drug therapy, computer-assisted

## Abstract

*Background and objectives*: The aim of this study is to describe the temporal change in alert override with a minimally interruptive clinical decision support (CDS) on a Next-Generation electronic medical record (EMR) and analyze factors associated with the change. *Materials and Methods*: The minimally interruptive CDS used in this study was implemented in the hospital in 2016, which was a part of the new next-generation EMR, Data Analytics and Research Window for Integrated kNowledge (DARWIN), which does not generate modals, ‘pop-ups’ but show messages as in-line information. The prescription (medication order) and alerts data from July 2016 to December 2017 were extracted. Piece-wise regression analysis and linear regression analysis was performed to determine the temporal change and factors associated with it. *Results*: Overall, 2,706,395 alerts and 993 doctors were included in the study. Among doctors, 37.2% were faculty (professors), 17.2% were fellows, and 45.6% trainees (interns and residents). The overall override rate was 61.9%. There was a significant change in an increasing trend at month 12 (*p* < 0.001). We found doctors’ positions and specialties, along with the number of alerts and medication variability, were significantly associated with the change. *Conclusions*: In this study, we found a significant temporal change of alert override. We also found factors associated with the change, which had statistical significance.

## 1. Introduction

Clinical decision support (CDS) plays a critical role in everyday clinical practice. Combined with advanced electronic medical records (EMRs), CDS is identified as a key for quality medicine, improving the safety and effectiveness of healthcare [1,2,3]. The use of CDS is associated with better adherence to clinical guidelines, a reduction in morbidity, and a reduction in medical errors [4,5,6,7]. These merits of CDS are influenced by various factors because the system is often integrated into complicated clinical settings [8,9]. A CDS to EMR is like an application to a smartphone, which is essentially intertwined with users and the work process.

The limitations of CDS, however, have been highlighted with an increase in CDS implementation. *Alert fatigue* is the most frequently addressed problem of CDS [10]. It is derived from *alarm fatigue*, which was observed during a study of aviation and nuclear fields where the false alarm rate was high [11]. Similar to these critical fields, healthcare providers tend to override alerts, which can lead to errors. Paradoxically, alert fatigue caused by safety stipulations established by the CDS can result in patient safety issues [12]. The effect of CDS on clinical outcomes seems as ambivalent.

Redesigning the CDS has been suggested to lower alert fatigue and alert override rates. Modals, i.e., *pop-ups*, are the least preferred way to increase the alert acceptance rate, whereas tailoring CDS for users is the best-suggested method [13]. Tiering alerts by severity or designing the alerts to be less interruptive seem to be a useful approach to increase the acceptance rate by users [14,15]. However, reports on minimally interruptive CDSs are extremely rare, and few studies have analyzed the various factors that affect the alert-override on the CDS based on a temporal trend [16]. Up to today, the effect of reducing interruptions on the acceptance of CDSs remains unanswered.

The aim of this study is to describe the temporal trend of an alert-override with a minimally interruptive CDS and analyze the factors associated with this change.

## 2. Methods

This is a retrospective study using data from an EMR. The study was approved by the institutional review board of the study site (IRB: 2019-05-038).

### 2.1. Study Setting

The study was conducted in an academic tertiary hospital in Seoul, which serves approximately two million out-patient (OPD) visits annually and provides an in-hospital service for 2000 beds. Approximately 1000 doctors and 6000 nurses work in such institutes. According to the governmental policy for tertiary hospitals, hospitals focus on care for critical and severe patients such as cancers and cardiovascular diseases.

### 2.2. Next-Generation Electronic Medical Record System and Medication Order (Prescription) System

**Figure 1:**
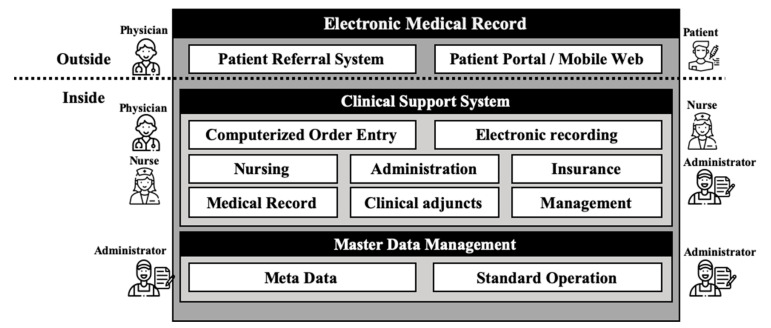
Overview of the electronic medical record (EMR) system of the study site. In addition to essential computerized order entry (CPOE), the EMR consists of various support systems such as patient portals and administrative functions.

The next-generation EMR used in this study was a home-grown system, which was launched in 2016 after almost three years of development. The system was a result of the “next-generation EMR project,” which was later named Data Analytics and Research Window for Integrated kNowledge (DARWIN). DARWIN is a comprehensive system that includes computerized order entry of the physicians as well as nursing, pharmacy, billing, research support, and even patient portal and web services. The conceptual architecture of DARWIN is described in Figure 1 [17].

**Figure 2:**
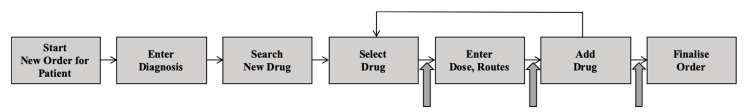
Medication order (prescription) sequence in electronic medical records. The process is carried out in a sequence, and the clinical decision support (CDS) in the study site intervened between certain action points. The points at which the CDS intervened are marked as thick gray arrows.

Within DARWIN, as well as other EMR systems, the medication process is carried out as a sequence of action triggered by physicians. Once the process is initiated, a patient is selected, the diagnosis is confirmed, and orders for tests and medications are followed. When ordering a specific medicine, the medicine is searched for, and specifics such as the routes, doses, and duration are entered. This sequence is usually repeated multiple times before the order is finalized. The CDS in this study is made up of activated sequences between drug selection and finalization. The process is also described in Figure 2.

### 2.3. Minimally Interruptive Clinical Decision Support Design

During the development of DARWIN, a minimally interruptive medication CDS was integrated into the system. A minimally interruptive CDS is similar to a non-interruptive CDS or is not likely to intervene in the work process of physicians. Although this type of alert may reduce alert fatigue, it may also result in decreased effectiveness of the CDS [18,19]. The CDS at the order entry is mainly for physicians and only covers medication alerts.

**Figure 3:**
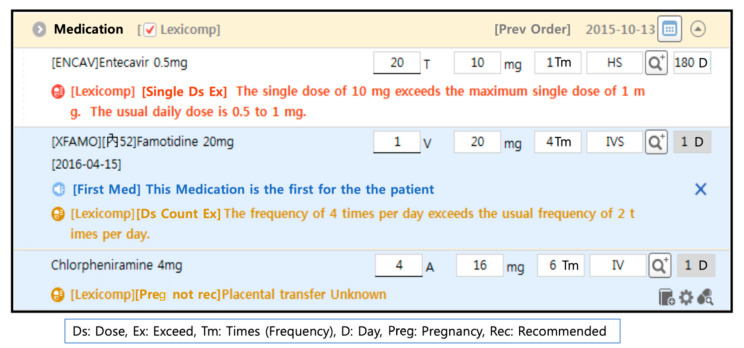
Description of clinical decision support (CDS) of the study. The system is designed as minimally interruptive; it does not block the user sites or stop the working process. Alert messages appear under the prescription line simultaneously while the order details are being inputted. The alert message disappears when the prescription details are revised according to the recommendation. The color represents the seriousness of the alerts: red, high; orange, medium; and blue, low.

The rule-based database for the CDS is supplied by Medi-Span (Wolters Kluwer Health, Philadelphia, PA, USA), with monthly updates. The types of alerts (domain) were age, allergy, disease, duplication, gender, lactation, pregnancy, dose, drug-drug interactions, and route. A screenshot of alerts along with the medication order is presented in Figure 3. During the study period, there was no significant change in the EMR and CDS. Only minor revisions and database updates had been made.

### 2.4. Study Participants

This study included patients from July 2016 to December 2017. Patients were included if they visited the out-patient department (OPD), emergency department (ED), or were admitted to inpatient wards (IW) during this 18-month period. Cases were selected if the ordering physicians had given orders throughout the study phase. If there had been any month during which no medication order was given by a doctor, the doctor was excluded from the dataset.

### 2.5. Data Extraction and Preparation

Medication order data were extracted from the clinical data warehouse (CDW) of the study site. Included data were the visit date, place of visit (OPD, ED, and IW), and medication (name, dose, and route). The specifics of the physicians were also collected: specialty department, position (trainee versus faculty (board-certified physicians)). CDS alert data were extracted from the DARWIN repository. Alert information contained the diagnosis code, reasons and types of alerts, and the level of caution.

### 2.6. Definition of Alert Override

Because the CDS is minimally interruptive, it does not mandate users to input response to the alert. Users may simply ignore the alert and continue the process. The definitions of an alert-override were as follows:

1. If neither medication was deleted from a drug-drug interaction (DDI), the DDI alert is overridden.
2. If a physician does not adjust the dose after a dose alert, the dose alert is overridden.
3. If a physician does not delete the medication order after an alert for age, allergy, disease, duplication, gender, lactation, pregnancy, or route, the alert is overridden.
4. This definition was validated in a previous study from the study site [16].

### 2.7. Data Analysis

The basic characteristics of the patients and alerts were described as simple statistics. A temporal change was demonstrated using time-series analysis. Piece-wise regression was conducted to determine the temporal change in the alert override rate. Finally, multivariate analysis was applied to determine if the change was statistically significant after adjusting for covariates. The data were analyzed using the statistical software R (v4.0.3) with the segmented (v1.3) package for piece-wise regression and the “lm” (linear model) function for linear regression.

For piece-wise regression, the alert override rate was analyzed using an iterative algorithm that utilizes maximum likelihood estimation of the observed data to identify unknown change point(s) in piece-wise regression models. The merit of using this method is that this method does not require prior knowledge of a changepoint. Changepoint estimate and 95% confidence intervals in the piece-wise regression model were calculated using this method.

## 3. Results

### 3.1. Basic Characteristics of Subjects

**Figure 4:**
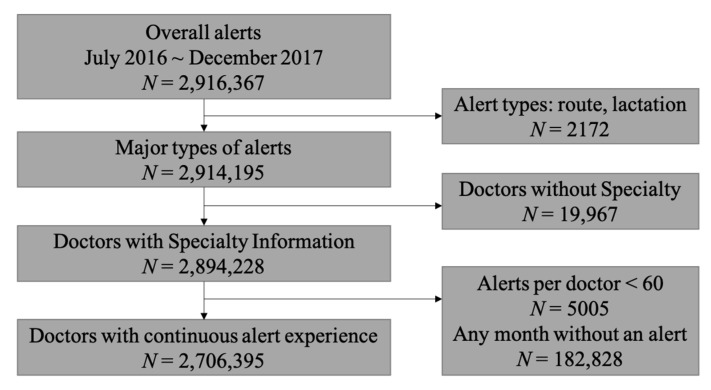
The alerts selection process of this study. All counts were based on alerts. Alerts were excluded if they were minor types or if alerts were fired to doctors whose experience on alerts was minimal (less than 60 during the study period). Alerts to doctors whose alert experience did not last 18 months were also excluded.

During the study period, 993 doctors wrote medication orders. Among the doctors, 37.2% were faculty (professors), 17.2% were fellows, and 45.6% were trainees (interns and residents). Doctors from every department were included; the most common specialty was general surgery (99% or 10.0%), general internal medicine (87% or 8.8%), and anesthesiology and pain medicine (85% or 8.6%), respectively. General internal medicine is a label for trainees in the internal medicine department, and its use is the reason why there were only one fellow and one faculty member in the department (Table 1) (Figure 4).

### 3.2. CDS Alerts and Override Information

During the study period, 2,706,395 alerts were fired to the subjects. Alerts were fired based on eight types of medication CDSs. Among these types, the dose type was the most common (1,551,876, or 57.3%), followed by age type (460,793, or 17.0%), and pregnancy type (292,266, or 10.8%). In terms of the location of the alert, OPD had a much larger number of alerts than IW or ED; it is also widely known that the patient and order volume was incomparably higher in OPD than in the other departments (Table 2).

Regarding the medication types, there were 1240 types of medications that caused an alert. The most common medication was ketorolac tromethamine (analgesics) (*n* = 106,851), which is one of the widely used non-steroidal anti-inflammatory drugs. The second and third most common drugs with an alerts were Propacetamol HCl (analgesics–nonnarcotic) (*n* = 78,432), and chlorpheniramine (antihistamines) (*n* = 75,361).

**Figure 5:**
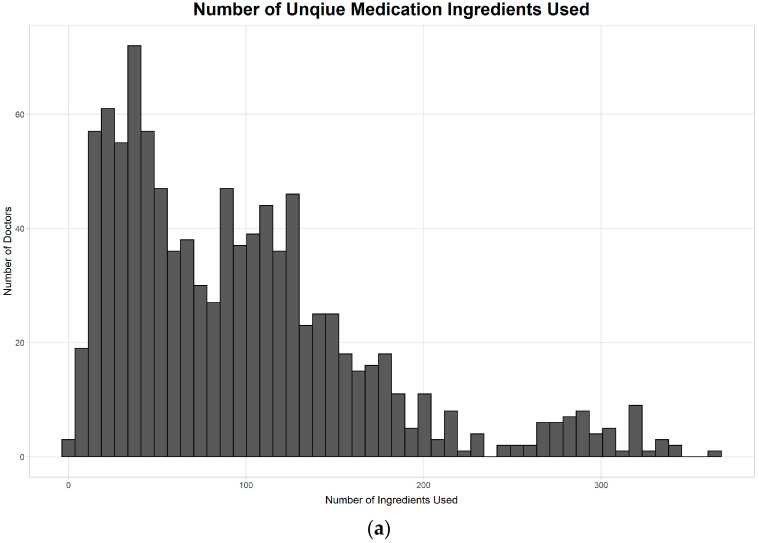

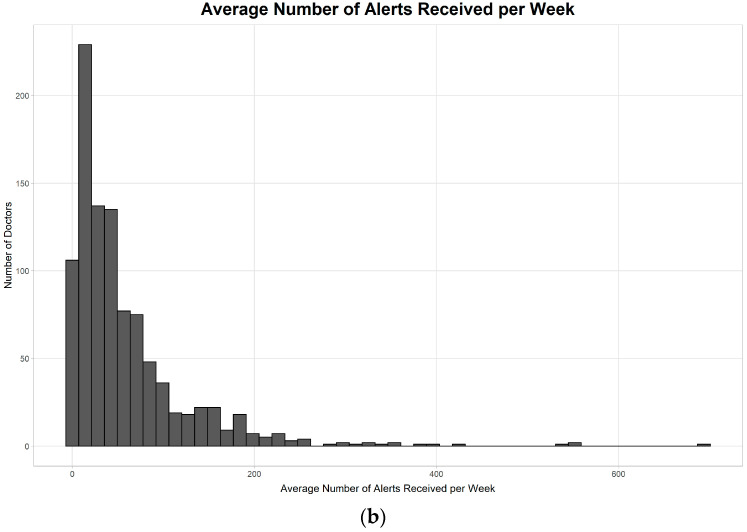
Number of medications used and alerts in the view of doctors. (**a**) The number of medication types by doctors; (**b**) The weekly number of alerts per doctor.

The types of medications ordered by doctors varied widely, whereas the median number was 84, with an interquartile range of 38 to 129. Moreover, the number of alerts per doctor also varied greatly. For the number of alerts per week, the median was 37.5, whereas the interquartile range was 15.1 to 74.7 during the 18-month period. The distribution of these numbers is demonstrated in Figure 5.

### 3.3. Temporal Change of Alerts and Alert Overrides

**Figure 6:**
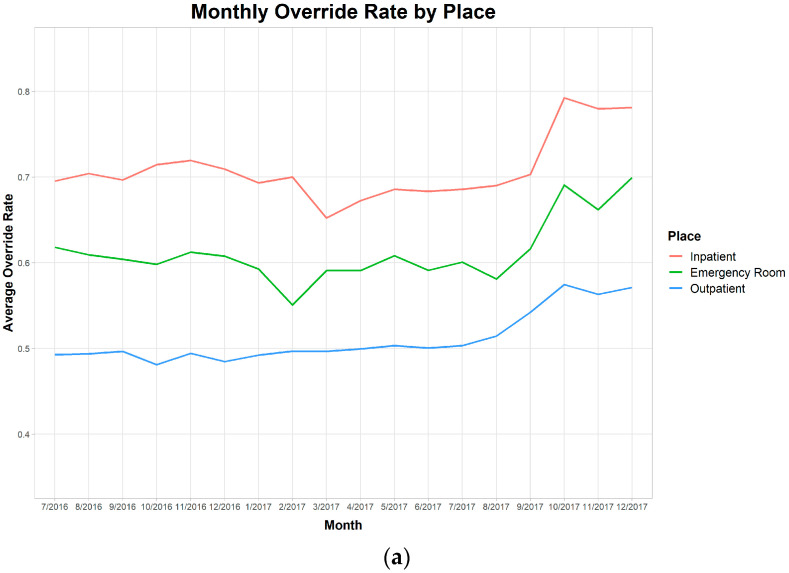

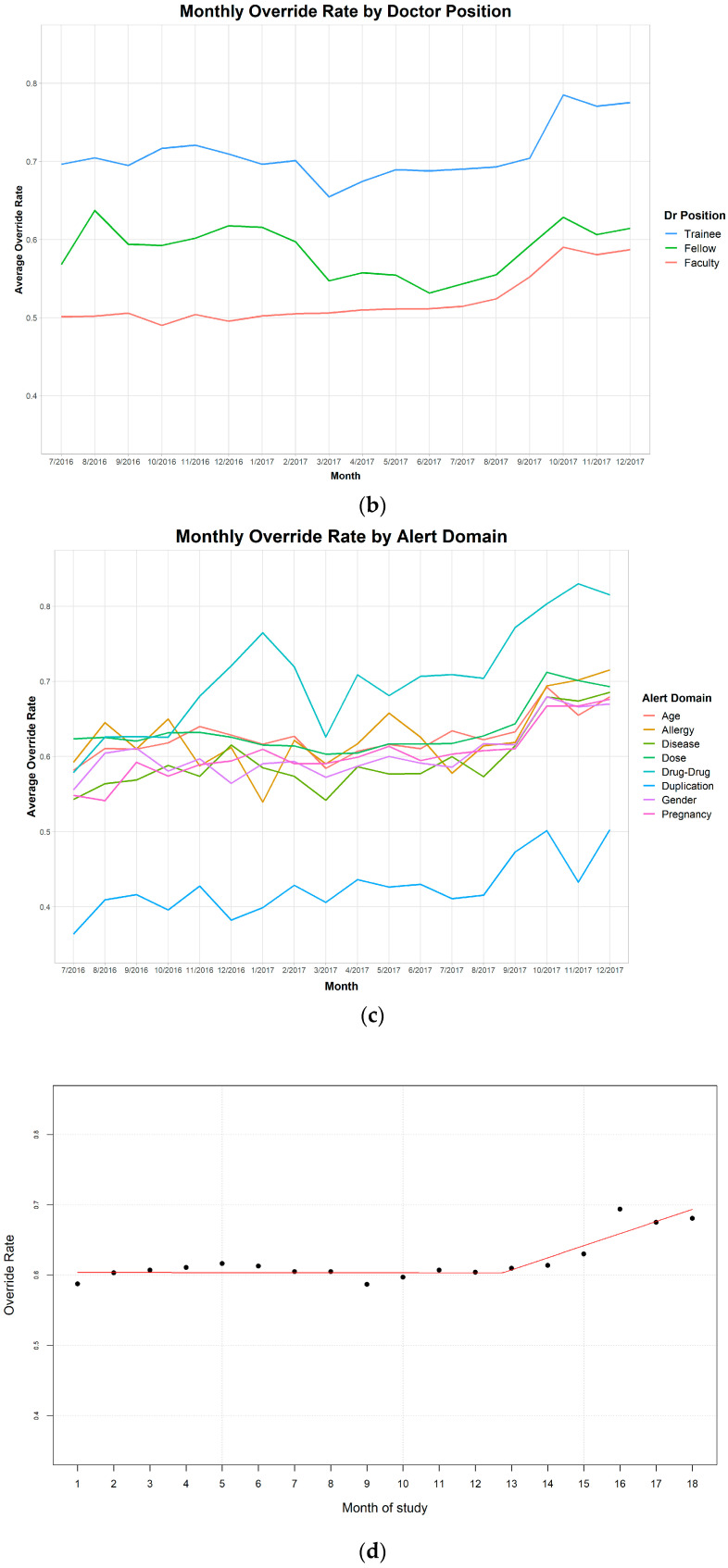
Trend of Alert firing rate and alert override rate by physicians. (**a**) Alert firing trend by place, (**b**) Alert firing by position; (**c**) Alert firing by CDS type; (**d**) The overall alert firing with piece-wise regression. These findings consistently indicate that there was a significant increase in the alert firing rate and override rate after 1-year of implementation.

To determine the change in trend regarding the alert overrides, a time-series analysis was conducted. The analysis was based on the place of order (OPD, IW, and ED) and position (faculty, fellow, and trainee) (Figure 6a,b). The trend of the alert overrides was also demonstrated based on the CDS type (Figure 6c). This analysis revealed that an increase in overrides was observed at an institution-wide level, not just by a few groups of doctors on a certain type. Piece-wise regression was used to determine the trend change, which confirmed that there was a significant change at month 12. (difference of slope: 0.017 Confidence Interval (0.010, 0.063)) (*p* < 0.001). The degree of change varied among the doctors (Figure 6d). The mean difference in the alert overrides between the first 12 months (before) and the last 6 months (after) was 4.9% (SD = 8.3%). The interquartile range was 1.5% to 9.3%.

### 3.4. Factors Associated with Increased Alert Overrides

Because the increase in alert overrides varied among doctors, a multivariate analysis was conducted to reveal the factors associated with an increase in alert overrides. The adjusted co-efficiency showed an increased override rate in the fellow and trainee groups compared to the faculty group. Surgeons showed less increase in override rates; other specialty doctors showed higher increases. When grouped into numbers of medication (how many types of drugs are prescribed by a doctor, i.e., drug variability), the higher the number was, the lower the alert overrides observed in the third quartile group. The frequency of received alerts showed an incremental effect on the override rate; groups with a higher number of alerts were associated with a greater change (Table 3).

## 4. Discussion

In this study, alert and alert-override patterns over an 18-month period were described. The study included all patients and providers across a tertiary hospital and found significant changes in alert override during the study period. We also found factors that were related to an increase in alert overrides. To the best of the authors’ knowledge, this is the first study to include inpatient, emergency department, and out-patient orders, which is important because doctors in tertiary centers tend to work for these sites simultaneously.

We also found a low rate of alert override, i.e., 61.9%, compared to previous reports of 49% to 94% [12,20,21]. This is even more impressive, considering that it has been widely accepted that CDS with a hard stop, which is a highly interruptive approach, is more effective [22]. The low rate of alert override may have resulted from the design of the CDS in which the relevant message is displayed at the bottom of the order line while the order is being typed in. The modification over message delivery, timing, and content adjustment are key components of a successful CDS [23,24].

The method used to determine the change in the trend of the override rate was piece-wise regression, also known as interrupted time-series analysis. The method is becoming increasingly used because of its robustness to controlling secular trends and ease of interpretation [25,26]. However, there remains a risk of producing misleading or incorrect outcomes if the statistical method is not properly used [27,28].

This study does not include the appropriateness of the alert overrides. Although there are many reasons for an alert override, it is difficult to determine the accurate reasons because many doctors do not always correctly reflect the reason for the override [29]. Overrides are often the result of the clinical context, which in turn is considered appropriate by chart reviews [30,31]. It is reasonable to consider that many of the alerts are appropriate, and the CDS can always be improved in terms of specificity, which means reducing the false-alert rate.

In this study, we found a statistically significant association between an override increase and factors specific to doctors. These factors were consistent with those of previous studies, which also included these factors. The specialty department, along with the number of medications and number of alerts, were reported as major causes of an alert override. Other studies have also suggested reasons for alert overrides, i.e., the clinical condition (severity) of the patients, [32] appropriateness of the medication, doctors’ habits, and admitting conditions of specialty departments [33,34].

Factors included as the place of order, types of alerts, and position of doctors who made the prescription orders could be considered as a static factor. Variables that could change over time, which include the degree of experience, the policies of the institution, or change of disease epidemiology, could be considered as temporal factors. These temporal factors’ association with alert override requires further studies since they were not covered in the scope of this study. A qualitative method could be appropriate since it could reveal situations that users had faced at that time.

The results of this study imply that even without intervention, minimally interruptive CDS alert overrides may increase after about one year of usage. This one year of usage may have made users of EMR familiar with the system; at the same time, it may have caused sufficient fatigue to increase the overrides. To prevent an override and make the system more effective, caution must be exercised by the high-risk group of doctors who can be defined by the results of this study. Medical doctors prescribing many different types of drugs, and receiving a large volume of alerts could be potential targets.

### Limitation

This study has certain limitations. First, the study was conducted in a single center. Because a CDS and an EMR, along with clinical norms, differ widely among institutions, the result of this study should be applied to other centers with caution. Second, this study did not cover all confounders of alerts in the study site. Alert fatigue may be driven by multiple sources, and other confounding factors could have influenced the outcome with varying degrees of interaction. Though there is no evidence, a significant change in the CDS rule engine could have influenced the outcome. Third, owing to the design of minimally interruptive CDSs, specific reasons for the overrides were unavailable. Without confirmed reasons for an alert override, it is difficult to determine the appropriateness.

## 5. Conclusions

In this study, we described the temporal trend of an alert-override with a minimally interruptive CDS and analyzed the factors associated with the change. The analysis revealed variables such as doctor’s specialty, frequency of prescription on specific drugs, and the number of alerts received as significant factors associated with the increase in the trend.

## Figures and Tables

**Table 1:** Composition of specialties among study subjects. General internal medicine is a specialty given to trainees in internal medicine, which resulted in only one staff member or fellow belonging to this category.

Department	Faculty *n* (%)	Fellow *n* (%)	Trainee *n* (%)	Total *n* (%)
General Surgery	35 (3.5)	9 (0.9)	55 (5.5)	99 (10.0)
General Internal Medicine	1 (0.1)	1 (0.1)	85 (8.6)	87 (8.8)
Anesthesiology and Pain Medicine	36 (3.6)	9 (0.9)	40 (4.0)	85 (8.6)
Pediatrics	19 (1.9)	16 (1.6)	32 (3.2)	67 (6.7)
Gynecology and Obstetrics	12 (1.2)	8 (0.8)	31 (3.1)	51 (5.1)
Neurology	21 (2.1)	11 (1.1)	12 (1.2)	44 (4.4)
Thoracic surgery	16 (1.6)	6 (0.6)	20 (2.0)	42 (4.2)
Gastroenterology	24 (2.4)	16 (1.6)	0 (0.0)	40 (4.0)
Orthopedic Surgery	13 (1.3)	6 (0.6)	20 (2.0)	39 (3.9)
Cardiology	24 (2.4)	9 (0.9)	0 (0.0)	33 (3.3)
Emergency Department	5 (0.5)	5 (0.5)	23 (2.3)	33 (3.3)
Hematology and Oncology	26 (2.6)	6 (0.6)	0 (0.0)	32 (3.2)
Ophthalmology	10 (1.0)	10 (1.0)	12 (1.2)	32 (3.2)
Neurosurgery	14 (1.4)	4 (0.4)	13 (1.3)	31 (3.1)
Otolaryngology	11 (1.1)	4 (0.4)	14 (1.4)	29 (2.9)
Psychiatry	8 (0.8)	5 (0.5)	16 (1.6)	29 (2.9)
Urology	12 (1.2)	5 (0.5)	12 (1.2)	29 (2.9)
Family Medicine	4 (0.4)	2 (0.2)	21 (2.1)	27 (2.7)

**Table 2:** Type of alerts and place of orders during the study period.

Alert Type	Place of Order			
	OPD *n*(%)	IW *n* (%)	ED *n* (%)	Total *n* (%)
Dose	584,414 (50.0%)	900,000 (62.8%)	67,462 (64.3%)	1,551,876 (57.3%)
Age	193,991 (16.6%)	248,050 (17.3%)	18,752 (17.9%)	460,793 (17.0%)
Pregnancy	164,644 (14.1%)	118,139 (8.2%)	9483 (9.0%)	292,266 (10.8%)
Disease	53,998 (4.6%)	74,781 (5.2%)	4171 (4.0%)	132,950 (4.9%)
Duplication	88,793 (7.6%)	15,096 (1.1%)	1540 (1.5%)	105,429 (3.9%)
Drug-Drug Interaction	39,801 (3.4%)	34,337 (2.4%)	1388 (1.3%)	75,526 (2.8%)
Gender	33,144 (2.8%)	22,614 (1.6%)	429 (0.4%)	56,187 (2.1%)
Allergy	10,411 (0.9%)	19,238 (1.3%)	1719 (1.6%)	31,368 (1.2%)

OPD, Outpatient Department; IW, Inpatient Ward; ED, Emergency Department.

**Table 3:** Factors associated with increased alert override by doctors. The outcome was defined as an incremental rate of alerts (delta) from the prior 12 months to the rest of the study phase.

Variables	Univariate Analysis			Multivariate Analysis		
	Coefficient	95% CI	*p*-Value	Coefficient	95% CI	*p*-Value
Doctor Position			<0.001			
Staff	Reference					
Fellow	0.14	(0.08, 0.19)	<0.001	0.53	(0.47, 0.58)	<0.001
Trainee	−0.92	(−0.94, −0.90)	<0.001	0.19	(0.17, 0.22)	<0.001
Doctor Specialty			<0.001			
Internal Medicine	Reference					
Surgical Department	−2.10	(−2.12, −2.07)	<0.001	−1.72	(−1.75, −1.70)	<0.001
Others	1.59	(1.57, 1.62)	<0.001	1.56	(1.53, 1.58)	<0.001
Order Drug Variability			<0.001			
1st quartile (low)	Reference					
2nd quartile	−2.59	(−2.61, −2.56)	<0.001	−2.37	(−2.40, −2.35)	<0.001
3nd quartile	−3.32	(−3.35, −3.30)	<0.001	−3.62	(−3.65, −3.60)	<0.001
4th quartile (high)	−1.39	(−1.42, −1.36)	<0.001	−2.31	(−2.35, −2.27)	<0.001
Volume of Alerts			<0.001			
1st quartile (low)	Reference					
2nd quartile	1.73	(1.70, 1.75)	<0.001	2.60	(2.58, 2.63)	<0.001
3nd quartile	1.40	(1.38, 1.43)	<0.001	2.91	(2.88, 2.94)	<0.001
4th quartile (high)	2.47	(2.44, 2.50)	<0.001	3.29	(3.26, 3.33)	<0.001
